# Detection of *Crenosoma* spp., *Angiostrongylus vasorum* and *Aelurostrongylus abstrusus* in Gastropods in Eastern Austria

**DOI:** 10.3390/pathogens9121046

**Published:** 2020-12-13

**Authors:** Hans-Peter Fuehrer, Simone Morelli, Julian Bleicher, Thomas Brauchart, Mirjam Edler, Nicole Eisschiel, Tatjana Hering, Sigrun Lercher, Karoline Mohab, Simon Reinelt, Theresa Stessl, Doris Fasching, Ricarda Nimphy, Anja Pelzl, Bita Shahi-Barogh, Licha Natalia Wortha, Karin Bakran-Lebl, Michael Duda, Helmut Sattmann, Roland Schaper, Donato Traversa, Anja Joachim

**Affiliations:** 1 Department of Pathobiology, Institute of Parasitology, University of Veterinary Medicine, 1210 Vienna, Austria; 1445144@students.vetmeduni.ac.at (J.B.); 1311869@students.vetmeduni.ac.at (T.B.); 1345189@students.vetmeduni.ac.at (M.E.); 1540629@students.vetmeduni.ac.at (N.E.); tatjana@heringweb.de (T.H.); 1441150@students.vetmeduni.ac.at (S.L.); karoline.mohab@gmx.at (K.M.); 1263337@students.vetmeduni.ac.at (S.R.); 1445113@students.vetmeduni.ac.at (T.S.); doris.fasching@gmail.com (D.F.); ricardanimphy@gmail.com (R.N.); P.ANJA.P@hotmail.com (A.P.); Bita.ShahiBarogh@vetmeduni.ac.at (B.S.-B.); licha.wortha@vetmeduni.ac.at (L.N.W.); karin.bakran-lebl@vetmeduni.ac.at (K.B.-L.); anja.joachim@vetmeduni.ac.at (A.J.); 2 Faculty of Veterinary Medicine, University of Teramo, 64100 Teramo, Italy; smorelli@unite.it (S.M.); dtraversa@unite.it (D.T.); 3 Department of Invertebrate Zoology, Natural History Museum Vienna, 1010 Vienna, Austria; michael.duda@nhm-wien.ac.at (M.D.); helmut.sattmann@nhm-wien.ac.at (H.S.); 4 Elanco Animal Health, 40789 Monheim, Germany; roland.schaper@elancoah.com

**Keywords:** *Angiostrongylus vasorum*, *Aelurostrongylus abstrusus*, *Crenosoma*, Austria, PCR, *Arion vulgaris*

## Abstract

Canine and feline cardiorespiratory parasites are of utmost relevance in veterinary medicine. Key epizootiological information on major pet metastrongyloids, i.e., *Angiostrongylus vasorum* and *Crenosoma vulpis* infecting dogs, and *Aelurostrongylus abstrusus* and *Troglostrongylus brevior* infecting cats, is missing from Austria. This study investigated their occurrence in 1320 gastropods collected in the Austrian provinces of Styria, Burgenland, Lower Austria, and in metropolitan Vienna. Metastrongyloid larvae were microscopically detected in 25 samples, and sequence analysis confirmed the presence of metastrongyloids in nine samples, i.e., *A. vasorum* in one slug (*Arion vulgaris*) (0.07%), *C. vulpis* in five slugs (one *Limax maximus* and four *A. vulgaris*) (0.4%), *A. abstrusus* in two *A. vulgaris* (0.17%), and the hedgehog lungworm *Crenosoma striatum* was detected in one *A. vulgaris*. The present study confirms the enzooticity of major cardiorespiratory nematodes in Austria and that canine and feline populations are at risk of infection.

## 1. Introduction

Cardiopulmonary metastrongyloid nematodes that affect dogs and cats are enzootic in Europe [1,2]. In the last years, these parasites have stimulated the interest of the veterinary scientific community for their emergence and clinical relevance [1,3,4]. Among them, *Angiostrongylus vasorum* and *Crenosoma vulpis* are the most relevant species infecting dogs [5,6], while *Aelurostrongylus abstrusus* and *Troglostrongylus brevior* are the most important species in cats [2,7].

*Angiostrongylus vasorum* (the “French heartworm”) infects the pulmonary arteries of dogs throughout Europe with a typical patchy geographical distribution, constituted by endemic foci with nearby low-prevalence areas [8]. In the last decade, *A. vasorum* has spread in various European regions, in both enzootic areas and areas previously free of infection [5,9,10]. Canine angiostrongylosis may be fatal and its clinical course is unpredictable, as it can be chronic, sub-clinic, acute, or hyperacute. A clinical diagnosis is almost impossible, as dogs infected with *A. vasorum* can show a plethora of nonspecific as well as cardiopulmonary, neurological, and gastrointestinal clinical signs [3,11,12].

*Crenosoma vulpis* (“fox lungworm”) lives in the bronchi, bronchioles, and trachea of dogs and other canids [13]. This nematode occurs at lower prevalence rates than *A. vasorum* in Southern Europe [8,14], while it is more prevalent in Central and Northern Europe [6,15]. Although dogs may display severe chronic cough, canine crenosomosis is rarely fatal [1,6].

Feline aelurostrongylosis caused by *A. abstrusus* (“cat lungworm”) is distributed worldwide. This nematode infects bronchioles and alveolar ducts of cats [16], which may be either subclinically infected or show respiratory or general clinical signs, for example, coughing, sneezing, wheezing, lethargy, depression, and occasionally, death [17,18,19].

During the past decade, *T. brevior* has increasingly been reported in domestic cats from Europe, mainly in countries of the Mediterranean Basin [20]. The natural host of *T. brevior* is the European wild cat *Felis silvestris,* but it may also infect domestic cats, causing severe infections especially in kittens and young animals, in which troglostrongylosis is often fatal [20].

All these cardiopulmonary nematodes have an indirect lifecycle, with gastropods acting as obligate intermediate hosts [1,20]. Surveys on the presence of metastrongyloid larvae in field-collected intermediate hosts is a useful approach for evaluating the occurrence of cardiopulmonary nematodes in a given area [21,22,23] and predicting/assessing the risk of infection for canine and feline populations [22,24]. In recent years, surveys on larval nematodes harbored by wild-caught mollusks have increasingly been performed, mostly in Europe [22,23,25] and South America [21,26]. Nevertheless, knowledge of the presence and distribution of cardiopulmonary nematodes in both definitive and intermediate hosts is still incipient and requires scientific refinements in certain European countries, such as Austria [22]. Given the great veterinary relevance of pet cardiopulmonary nematodes and the apparent geographical expansion of extraintestinal parasitoses of dogs and cats due to various factors [8,16,24,27], the present study investigated the occurrence of major canine and feline metastrongyloids in snails and slugs in Austria.

## 2. Results

**Figure 1:**
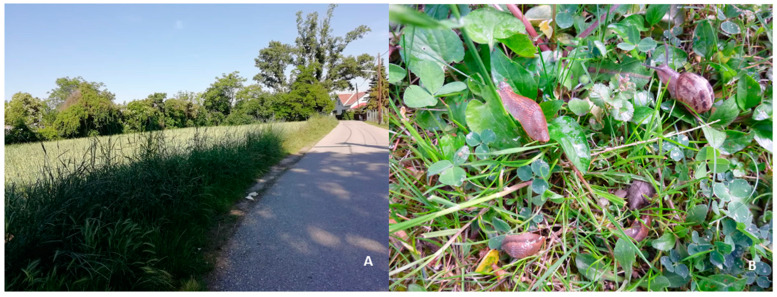
Sampling sites of gastropods in Eastern Austria. (**A**) Vienna; (**B**) Gerasdorf.

Overall, 1320 gastropods belonging to 12 different species were collected (Figure 1 and Table 1). Among those, 964 (73%) were slugs. Among all gastropods, 78% represented *Arion vulgaris* and *Cornu aspersum*.

**Figure 2:**
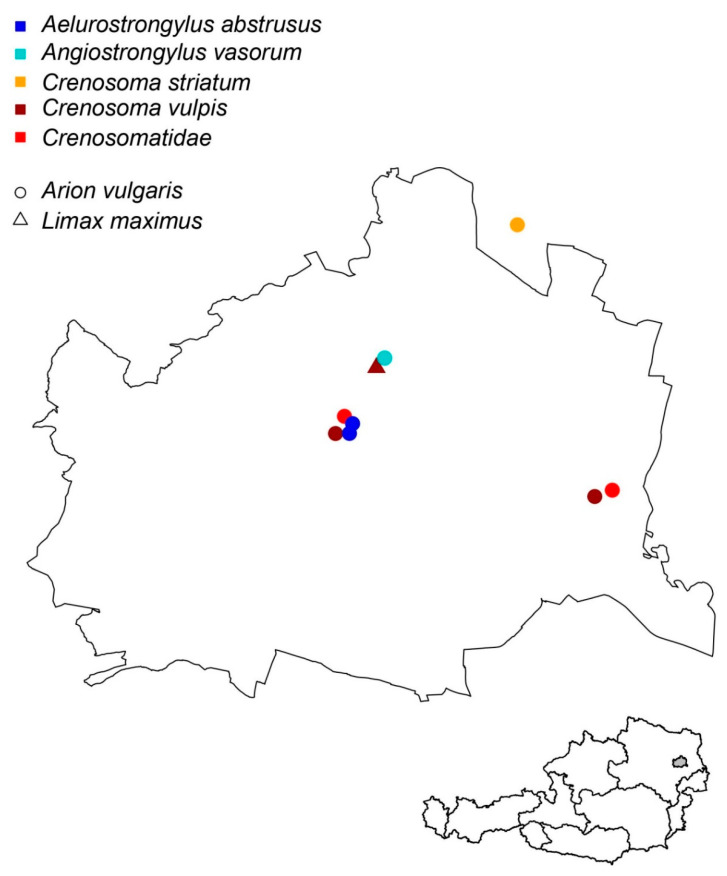
Collection sites of lungworms in Eastern Austria (metropolitan Vienna and its surroundings).

Nematodes were microscopically documented in 710 (53.8%) gastropods, and metastrongyloid larvae were found in 25 individual specimens. The molecular analysis provided a sound sequence confirmation of nine samples (Table 2). Positive specimens were collected in Vienna (*n* = 8) and in Gerasdorf/Lower Austria, directly bordering Vienna (Figure 2). Overall, six (0.45%) and two (0.15%) gastropods contained canine or feline metastrongyloids, respectively (Supplementary Materials MT757393, MT757394, MT758698, and MT758699).

*Angiostrongylus vasorum* was documented in one Spanish slug (*A. vulgaris*; 0.07%) collected on the Danube Island, a recreational area in Vienna highly frequented by dogs but also populated by foxes. *A. abstrusus* was found in two Spanish slugs (0.15%) sampled in a park in Central Vienna (Friedensbrücke), while *C. vulpis* was recorded in five (0.4%) slugs (i.e., four *A. vulgaris* and one *Limax maximus*) collected in areas for dog runs, recreational areas, and the periphery of Vienna. The sequence of *C. striatum* (a parasite of hedgehogs) was confirmed in one *A. vulgaris* (0.07%) collected in a private garden in Gerasdorf (Lower Austria).

## 3. Discussion

The present data demonstrated the occurrence of canine and feline cardiopulmonary nematodes in intermediate gastropod hosts from Austria. In addition, the first natural infection of *L. maximus* by *C. vulpis,* which had already been experimentally shown [28], was demonstrated.

Overall, the infection rates, found herein, are lower as compared with previous data from Austria [22] and data obtained in epizootiological studies carried out in other European countries, for example, in Denmark [29], Poland [30], Scotland [31], UK [24,32], Germany [25], and Greece [23]. This discrepancy could be due to different reasons. Intrinsic hindrances exist in the molecular analysis of gastropods, for example, the presence of mucopolysaccharides co-precipitating with DNA and inhibiting the activity of DNA polymerase [33]. Furthermore, it should be considered that, in other studies, gastropods were collected from known enzootic/hyperenzootic areas for *A. vasorum*, for example, Denmark, Scotland, UK, central Germany [24,25,29,31,32], or for *A. abstrusus*, i.e., Greece [23].

The lower percentage rates, herein obtained, as compared with a recent study performed in Austria [22] could depend on the following: (i) the bigger sample size examined in the present study, which probably allowed a more accurate estimation of the true prevalence of canine and feline metastrongyloids from Austria; (ii) the abovementioned inhibition of the DNA polymerase that could have affected the present results, leading to a possible underestimation. Indeed, 14 snails harbored metastrongyloid larvae for which a species diagnosis was not possible at microscopic examination. It cannot be excluded that they belonged to species of veterinary interest, despite a negative PCR result. However, according to these data, Austria currently appears to be a low-enzooticity area for pet respiratory metastrongyloids.

A seasonal trend with higher presence in gastropods during autumn has been suggested for *A. vasorum* [24] and *C. vulpis* [25]. This may further account for the low prevalence obtained here as compared with those from a large-scale survey conducted in Germany [25] where, as in this study, gastropods were collected from late spring to early autumn. However, it should be considered that relatively high prevalence rates of *A. vasorum* were also detected in slugs during the summer [25]. For this reason, possible seasonality patterns should be investigated in more detail in order to ultimately assess whether different prevalence rates can be found in gastropods collected in different seasons.

Canine angiostrongylosis and crenosomosis have seldom been described in Austria in the past. Two previous published cases of dogs infected with *A. vasorum* were considered to be imported. Of these, one dog repeatedly travelled to the high-enzootic Southern France [34]. In the other case, the dog was born in Corse, and then brought to Austria [35]. Although *A. vasorum* infections have never been documented in Corse, this parasite is most probably present in the island, as it is enzootic in Sardinia [36], continental Italy, and France [8,37]. Nevertheless, an autochthonous origin of the reported infections cannot be excluded, as this parasite is enzootic in almost all countries bordering Austria [5,38] and cases have been reported sporadically from veterinary practitioners in the past years (Barbara Hinney, personal communication). Regardless of whether *A. vasorum* was imported in Austria or not, the retrieval of *A. vasorum* larvae in intermediate hosts collected in this study confirms that dogs living in Austria are at risk of infection with this parasite. Although *C. vulpis* can occur with high prevalence among foxes in Austria [39,40] and circulates among intermediate host populations (as shown in the present study), to the best of our knowledge it has only recently been detected in Austrian dogs for the first time [41].

The enzooticity of *A. abstrusus* in intermediate hosts in Austria (data of the present study [22]) is in accordance with documented cases of cat aelurostrongylosis in Austria (Barbara Hinney, personal communication). This nematode is enzootic in cat populations throughout Europe, including countries neighboring Austria [16,42]. Therefore its occurrence was also expected in Austrian cats, although it was not found in a recent survey [2].

In the present study, *T. brevior* was not been found in snails, although it has been recorded very recently in mollusks collected in a recreational area in Vienna [22]. The natural life cycle of this crenosomatid is strictly related to the European wildcat (*Felis silvestris*) [20] that lives in forested environments [43]. Wildcats are present in the Austrian territory especially in eastern areas of the country [44]. Therefore, the presence of *T. brevior* could be expected in felines living in Austria. However, although its recent detection in gastropods in Vienna [22] might suggest its circulation in Austrian felines, the significance of this finding is controversial. In fact, *T. brevior* has not been described in domestic or in wild cats from Austria, and further investigations are necessary to determine its presence in final hosts in this area.

Overall, knowledge on canine and feline cardiorespiratory metastrongyloids is still scarce, and precise epizootiological pictures still have to be drawn by geographic area. At present, it cannot be determined with certainty if their rarity in Austria is due to particular biological or phenological drivers or if they have been poorly investigated in the past. However, the fact that the vast majority of infected gastropods in this study belonged to *A. vulgaris*, an invasive species to Austria [45], indicates that the number of respiratory metastrongyloids infections in dogs and cats could rise in the near future. This hypothesis is corroborated by the growth of the fox population in Austria [46]. Indeed, foxes are natural reservoirs for both *A. vasorum* and *C. vulpis* [47,48] and it cannot be excluded that the growing and expanding fox population may have introduced these parasites to the country, and therefore could be responsible for the apparent emergence of canine metastrongyloids in Austria.

In conclusion, Austrian veterinary practitioners are herein called to increase their awareness of parasitoses caused by canine and feline metastrongyloids, which should be considered in the differential diagnosis in dogs and cats presented with cardiorespiratory signs.

Finally, the potential impact of *A. vasorum* and *C. vulpis* in dogs and of *A. abstrusus* and *T. brevior* in cats requires future studies that aim at evaluating their occurrence in Austrian canine and feline populations.

## 4. Materials and Methods

### 4.1. Snail Collection and Digestion

**Figure 3:**
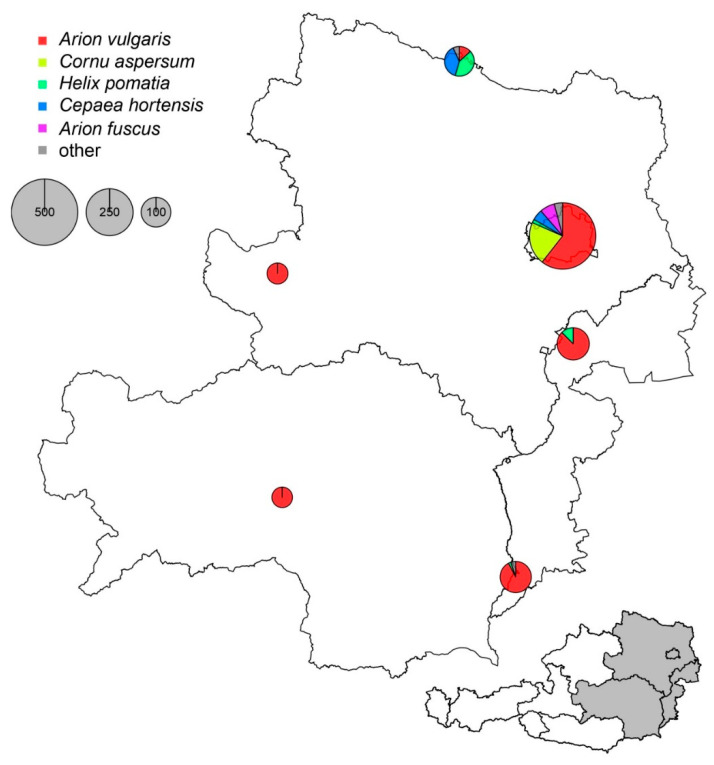
Collection sites and distribution of gastropods in Eastern Austria.

Gastropods (slugs and snails) were collected in the Austrian provinces of Styria, Burgenland, Lower Austria, and metropolitan Vienna (Figure 1 and Figure 3) from April to October 2019. The focus was on gastropods known to be competent vectors of canine lungworms (such as the invasive species *A. vulgaris* and *C. aspersum*), and on areas where dogs, cats, and wild carnivores are frequently observed (e.g., urban and suburban dog parks). An Eastern Austrian gastropod atlas (https://www.vetmeduni.ac.at/schnecken-atlas/), clearly indicating which species could be sampled, had been prepared prior to the sampling period and was used to prevent collection of protected species. Scientists with governmental sampling permissions (RU-BE-64/020-2019 and ABT13-53W-50/2018-2) collected specimens of the protected species *Helix pomatia*. After collection, live gastropods were transferred to the Natural History Museum Vienna for identification and verification of species, then, cryo-euthanized at the Institute of Parasitology, Vetmeduni Vienna, and stored at −20 °C until further processing. Prior to digestion, a small pinhead-sized part of the gastropod head was clipped off and stored at −20 °C for further molecular species verification with barcoding of the mt COI gene. Gastropods were digested with hydrochloric acid/pepsin, as described previously [21,25] and sediments were transferred to petri dishes for microscopic examination under a stereomicroscope (Olympus SZH10 Research Stereo Microscope, Olympus Austria, Vienna, Austria). Nematode larvae from each positive sample were transferred to microscopic slides for digital photo analysis (Olympus Provis AX70 and cellSens, Olympus, Austria), and identified according to morphologic keys available in the literature [49,50,51,52]. Slides and remaining sediments were stored at −20 °C for molecular analysis.

### 4.2. Molecular Analysis

The sediments of gastropod samples positive for nematode larvae were further analyzed using molecular techniques. Furthermore, DNA was also extracted from all larvae on slides that were microscopically identified as members of the Metastrongyloidea. DNA was extracted with a DNeasy Blood and Tissue Kit (Qiagen, Hilden, Germany), according to the manufacturer’s instructions. Samples were preliminarily screened with nested PCRs targeting the ribosomal ITS regions of bursate nematodes using primers NC1/NC2 for Nest 1 and NC1/MetR for Nest 2, as reported previously [53,54]. PCRs were carried out in a final volume of 25 μL using 5× Green Reaction Buffer and GoTaq G2 Polymerase (5 U/µL; Promega, Germany). PCR products were visualized by electrophoresis on 2% agarose gels stained with Midori-Green Advance^®^ (Biozym, Hessisch Oldendorf, Germany). Because of the limited number of positive results obtained with the conventional nested PCR, the samples were also screened with a high-fidelity polymerase using the GoTaq^®^ Long PCR Master Mix (Promega, Madison, USA) to obtain longer amplicons. The cycling conditions were identical to those for the conventional nested PCR. Then, the samples were subjected to PCRs specific for the mitochondrial 12S rRNA gene (primers Fila-12SF and Fila-12SR) and for the nuclear 18S rRNA gene (primers NC18SF1 and NC5BR) [55]. The obtained PCR products were subsequently sequenced at LGC Genomics GmbH, Germany.

## Figures and Tables

**Table 1:** Gastropods collected in Eastern Austria.

Species	n	%	Province
*Arion vulgaris*	882	66.8%	V, LA, ST, B
*Cornu aspersum*	150	11.4%	V, LA
*Helix pomatia*	96	7.3%	V, LA, ST, B
*Cepaea hortensis*	93	7%	V, LA
*Arion fuscus*	52	3.9%	V
*Limax maximus*	26	2%	V, LA
*Arianta arbustorum*	11	0.8%	LA
*Cepaea nemoralis*	3	0.2%	ST
*Limax cinereoniger*	3	0.2%	ST
*Causacotachea vindobonensis*	2	0.1%	ST
*Arion fasciatus*	1	<0.1%	LA
*Macrogastra ventricosa*	1	<0.1%	V

V, Vienna; LA, Lower Austria; ST, Styria; B, Burgenland. Specimens belonged to the genera *Arion* and *Limax* are slugs, the other genera are snails.

**Table 2:** Sequence confirmed lungworm larvae in gastropods in Eastern Austria.

Species	Host	Collection Site (Province)	Max. % Identity to GenBank Entries	GenBank ID
*Angiostrongylus vasorum*	*Arion vulgaris*	Danube Island (V)	99.8% (EU627597) UK, dog	MT757393
*Aelurostrongylus abstrusus*	*Arion vulgaris*	Friedensbrücke (V)	100% (JX519458), cat	MT758698
*Aelurostrongylus abstrusus*	*Arion vulgaris*	Friedensbrücke (V)	100% (JX519458), cat	nd
*Crenosoma striatum*	*Arion vulgaris*	Gerasdorf (LA)	100% (KR868716), Germany, hedgehog	MT757394
*Crenosoma vulpis*	*Limax maximus*	Danube Island (V)	100% (KR920039), Italy, fox, dog	MT758699
*Crenosoma vulpis*	*Arion vulgaris*	Donaustadt (V)	100% (KF836608), Germany, red fox	nd
*Crenosoma vulpis*	*Arion vulgaris*	Donaustadt (V)	100% (KF836608), Germany, red fox	nd
*Crenosoma vulpis*	*Arion vulgaris*	Friedensbrücke (V)	99.6% (KR920039), Italy, fox, dog	nd
*Crenosoma vulpis*	*Arion vulgaris*	Friedensbrücke (V)	100% (KF836608), Germany, red fox	nd

V, Vienna; LA, Lower Austria; A, Arion, L, Limax; nd, not determined/sequence too short for GenBank^®^ submission.

## Supplementary Materials

Sequences (accession numbers: MT757393, MT757394, MT758698, and MT758699) are available at NCBI.
